# Use of red, far-red, and near-infrared light in imaging of yeasts and filamentous fungi

**DOI:** 10.1007/s00253-022-11967-2

**Published:** 2022-05-23

**Authors:** István Pócsi, Zsuzsa M. Szigeti, Tamás Emri, Imre Boczonádi, György Vereb, János Szöllősi

**Affiliations:** 1 Department of Molecular Biotechnology and Microbiology, Institute of Biotechnology, Faculty of Science and Technology, University of Debrecen, Egyetem tér 1, 4032 Debrecen, Hungary; 2 Department of Biophysics and Cell Biology, Faculty of Medicine, University of Debrecen, Egyetem tér 1, 4032 Debrecen, Hungary; 3 MTA-DE Cell Biology and Signaling Research Group, Faculty of Medicine, University of Debrecen, Egyetem tér 1, 4032 Debrecen, Hungary; 4 Faculty of Pharmacy, University of Debrecen, Egyetem tér 1, 4032 Debrecen, Hungary

**Keywords:** Phototoxicity, Light sensing, Fluorescent proteins, Imaging toolboxes, Live-cell imaging, High-resolution microscopy, Time-lapse microscopy

## Abstract

**Abstract:**

While phototoxicity can be a useful therapeutic modality not only for eliminating malignant cells but also in treating fungal infections, mycologists aiming to observe morphological changes or molecular events in fungi, especially when long observation periods or high light fluxes are warranted, encounter problems owed to altered regulatory pathways or even cell death caused by various photosensing mechanisms. Consequently, the ever expanding repertoire of visible fluorescent protein toolboxes and high-resolution microscopy methods designed to investigate fungi in vitro and in vivo need to comply with an additional requirement: to decrease the unwanted side effects of illumination. In addition to optimizing exposure, an obvious solution is red-shifted illumination, which, however, does not come without compromises. This review summarizes the interactions of fungi with light and the various molecular biology and technology approaches developed for exploring their functions on the molecular, cellular, and in vivo microscopic levels, and outlines the progress towards reducing phototoxicity through applying far-red and near-infrared light.

**Key points:**

•* Fungal biological processes alter upon illumination, also under the microscope*

•* Red shifted fluorescent protein toolboxes decrease interference by illumination*

•* Innovations like two-photon, lightsheet, and near IR microscopy reduce phototoxicity*

## Introduction

It is an outstanding feature of the creatures belonging to the Kingdom of Fungi that they can sense light (Idnurm et al. 2010; Molin et al. 2020) and filamentous fungi can even distinguish between colours owing to their versatile photoreceptors (Yu and Fischer 2019; Yu et al. 2020, 2021). Not surprisingly, these organisms respond to incident light adequately and this response will affect nearly all aspects of fungal life (Yu and Fisher 2019). Excessive light elicits stress response as well, and vivid communication with various stress sensing, signalling, and stress response pathways have been elucidated (Fuller et al. 2013; Yu et al. 2016, 2019, 2021; Bodvard et al. 2017; Igbalajobi et al. 2019; Molin et al. 2020). Well-documented overlaps with oxidative stress responses (Chen et al. 2009; Fuller et al. 2013, 2015; Molin et al. 2020; Tagua et al. 2020) clearly indicate the generation of harmful reactive oxygen species (ROS) alarming oxidative stress defence especially at short wavelengths (Fuller et al. 2015; Igbalajobi et al. 2019).

Importantly, increasing the toxic effects of light on fungi is the primary aim in the development of novel tools to control fungal growth by illumination. For example, the growth inhibitory effects of extensive light can be further accelerated by concomitantly exposing fungal cells to photosensitizing agents, which have led to the development of various antifungal photodynamic therapies (Rodrigues et al. 2020; Shen et al. 2020; Rodríguez-Cerdeira et al. 2021).

Nevertheless, the very same phototoxicity phenomenon can be detrimental especially when prolonged time-lapse imaging techniques are applied in order to visualize cellular and subcellular structures and dynamics in living fungal cells (Frigault et al. 2009). The development of novel fluorescent reporter proteins with excitation/emission wavelengths in the red, far-red, and near-infrared spectra may ameliorate phototoxicity in time-dependent long-exposure microscopic techniques (Bialecka-Fornal et al. 2016; Icha et al. 2017). This approach may require the elaboration of new technical setups with higher intensity illumination and/or increased camera exposure time (Frigault et al. 2009; Mubaid and Brown 2017). The high translucency of host tissues towards red, far-red, and infrared light in animal-fungus and plant-fungus interaction studies has also fuelled the development of new long-wavelength reporting protein systems in a number of fungal species (Leal Jr. et al. 2010; Su et al. 2018; Vallarino et al. 2018). In addition, red, far-red, and infrared light have been used successfully to study host-fungus interactions deep in tissues (Bruns et al. 2010; Hasenberg et al. 2011; Sørensen et al. 2012; Vasilchenko et al. 2016; Lee et al. 2018). Combining red-shifted fluorophores with two-photon microscopy afforded increased spatial resolution in addition to better penetration (Tolić-Nørrelykke et al. 2004; Lemar et al. 2005, 2007; García-Marcos et al. 2008; Váchová et al. 2009).

In time-lapse imaging, proper selection of excitation light, exposition frequency, and time, as well as fluorescence marker proteins, may help minimize phototoxicity (Escorcia et al. 2019). Future tendencies may include the application of near-infrared fluorescent reporter proteins in fungi (Wosika et al. 2016) and the use of diodes emitting light in the infrared spectrum, e.g. at *λ* = 940 nm to monitor fungal growth (Nagy et al. 2014; Talas et al. 2019). Here, we provide an overview of fungus-light interactions, the exploitation and minimization of phototoxicity in fungal cultures, the development of assays based on fluorescent reporter proteins as well as the tendency of shifting the wavelengths used towards the red, far-red, and near-infrared. Overall, the accumulated evidence argues for the more widespread application of far-red and infrared microscopic techniques in the daily routine of mycologists.

## Fungi and light

Filamentous fungi cannot only sense the presence and absence of light but they are able to detect differences in the intensity and wavelength of light and even sense its direction (Corrochano and Galland 2016; Yu and Fischer 2019). Most filamentous fungi evolved complex and sophisticated light sensing systems, consisting of blue-, green-, and red-light photoreceptors (Idnurm et al. 2010; Yu et al. 2020). The blue-light photoreceptors include the well-characterized WC and MAD proteins (Froehlich et al. 2002; Silva et al. 2006; Sanz et al. 2009) and also some less known blue-light photoreceptors like photolyases/cryptochromes and BLUF (“Blue Light Using Flavin”) proteins (Berrocal-Tito et al. 2007; Bayram et al. 2008; Brych et al. 2016; Cohrs and Schumacher 2017). Opsins sense green light (García-Martínez et al. 2015) while phytochromes respond to red and far-red light (Blumenstein et al. 2005; Froehlich et al. 2005). For example, putative phytochrome (FphA), WC-1 (LreA), opsin (NopA), and cryptochrome (CryA) orthologues have been identified in the genome of *Alternaria alternata*, a common plant pathogen and post-harvest contaminant fungus (Igbalajobi et al. 2019).

Light can inform fungi on their orientation, e.g. whether they are growing on the ground or below that, or on the expected or unexpected changes in their environment, e.g. intensity of UV radiation, temperature, or consequences of elevated temperature such as decreased humidity. Genome-wide transcription studies demonstrated that large groups of genes are controlled by this important environmental factor (Rosales-Saavedra et al. 2006; Chen et al. 2009; Fuller et al. 2013; Bayram et al. 2016; Tagua et al. 2020; Yu et al. 2020, 2021). Light of different wavelengths can regulate germination, vegetative growth, asexual and sexual development, stress responses, pathogenic or symbiotic relationships, metabolism including mycotoxin production and circadian rhythm in fungi (Ruger-Herreros et al. 2011; Yu and Fischer 2019; Díaz and Larrondo 2020; Schumacher and Gorbushina 2020), and these observations should always be taken into consideration in any illuminated fungal cultures.

In addition to light informing fungi of impending stresses, light itself can also be a stressor for them. The near UV-blue spectrum can induce DNA damages, while the far-red spectrum can lead to heat stress (Fuller et al. 2013, 2015; Tagua et al. 2020). Moreover, a slight reduction of growth in light-exposed fungi is considered a general phenomenon, which can be coupled to the formation of ROS elicited by the short wave components of the visible light (Fuller et al. 2015). In addition to oxidative damages, a number of metabolic pathways are light responsive in fungi (Fuller et al. 2013), which may also be disadvantageous for growth.

It is noteworthy that only few ascomycetous yeasts contain any homologs of dedicated photoreceptors commonly found in most filamentous fungi (Idnurm et al. 2010; Okamoto et al. 2013). Nevertheless, components of visible light can interact with porphyrins and flavins in the baker’s yeast *Saccharomyces cerevisiae* causing extensive cytochrome damage (Robertson et al. 2013) and the generation of H_2_O_2_ by flavin-containing oxidases like Pox1 peroxisomal acyl-CoA oxidase (Bodvard et al. 2017; Molin et al. 2020). As a consequence, the activity of important protein kinases like the cAMP-dependent protein kinase A and the Hog1 mitogen-activated protein kinase (MAPK) is modulated and the stress response transcription factors Msn2/4 translocate to the nuclei to combat light-induced stress in yeast cells (Bodvard et al. 2017; Molin et al. 2020). In addition, the transcriptions of genes coding for key elements of the oxidative stress defence system like *TRX2* encoding cytoplasmic thioredoxin are upregulated (Robertson et al. 2013).

In filamentous fungi, several signalling pathways are also under the control of light. FphA phytochrome-dependent light signalling activates SakA/HogA MAPK in *A. nidulans* hyphae, where this MAPK pathway responds to various environmental stress stimuli (Yu et al. 2016). Similar FphA-dependent nuclear accumulation of the *A. alternata* HogA orthologue has also been observed (Igbalajobi et al. 2019). Interestingly, TcsB hybrid kinase and FphA phytochrome seem to be responsible for the temperature sensing of *A. nidulans* and this environmental signal is channelled into the SakA/HogA MAPK pathway (Yu et al. 2019). In *Aspergillus fumigatus*, LreA and FphA blue and red light receptors act together in the white light to induce tolerance of oxidative stress elicited by H_2_O_2_ (Fuller et al. 2013). At the same time, elimination of either FphA or LreA results in an increased oxidative stress tolerance of *A. alternata* owing to the upregulation of genes coding for oxidative stress defence enzymes (catalase, superoxide dismutases) (Igbalajobi et al. 2019).

All these observations indicate that both yeasts and filamentous fungi are well-armoured to cope with stresses either predicted or induced by light.

## Incorporation of red, far-red, and near-infrared fluorescent proteins into imaging toolboxes

The application of fluorescent proteins, highlighters, fluorescent probes, and biosensors is part of the daily routine today in any cell biological laboratory and needs the continuous monitoring of cell death processes to avoid cellular photodamages especially when UV-light excitation is employed (Frigault et al. 2009). Although eukaryotic cells including yeasts and filemantous fungi possess a remarkable capability to defend themselves against the deleterious effects of high intensity illumination, proper selection of equipment, image acquisition platforms and accessories, and optimal settings are necessary to avoid phototoxic effects of either transmitted light as well as that of excitation light in the fluorescence microscopy of these cells (Frigault et al. 2009; Magidson and Khodjakov 2013; Icha et al. 2017; Laissue et al. 2017; Ojha and Ojha 2021). One obvious option to solve this problem could be to limit the formation of ROS by reducing O_2_ partial pressure in the cultures and/or to increase the resilience of cells against light stress and concomitant oxidative stress by the addition of antioxidants like Trolox (Douthwright and Sluder 2017). Nevertheless, reducing O_2_ and supplementing fungal cultures with antioxidants are likely to induce significant, unwanted morphological and physiological changes (Emri et al. 2004; Raspor et al. 2005; Barker et al. 2012; Kowalski et al. 2019).

Selecting red, far-red, or near-infrared light for excitation may also decrease phototoxicity but may require higher intensity illumination to get satisfactorily bright images (Bialecka-Fornal et al. 2016; Icha et al. 2017), which may cause localized heating (Frigault et al. 2009; Mubaid and Brown 2017). Reducing excitation light intensity with a concomitant increase in the camera exposure time may attenuate phototoxicity especially in long-term time-lapse imaging of living cells (Mubaid and Brown 2017). The use of red-shifted wavelengths in super-resolution microscopy may also be applicable for reducing phototoxicity (Tosheva et al. 2020). In addition, the high translucency of animal tissues to far-red and near-infrared light has enforced the development of novel fluorescent proteins, which are excited and emit light in this light range (Olenych et al. 2007; Filonov et al. 2011; Piatkevich and Verkhusha 2011; Miyawaki et al. 2012; Piatkevich et al. 2013; McIsaac et al. 2014; Bindels et al. 2016; Yu et al. 2016; Rodriguez et al. 2016; Ding et al. 2018; Hou et al. 2019a, b; Wu et al. 2021).

### Red fluorescent proteins in use

Red fluorescent proteins can be divided into three groups based on their emission maxima: orange (*λ*_max_ = 550–590 nm), red (*λ*_max_ = 590–630 nm), and far-red (*λ*_max_ > 630 nm in the visible spectrum of light) (Piatkevich and Verkhusha 2011; Miyawaki et al. 2012). Some of these red-shifted fluorophores are employed routinely for tagging proteins in Mycology (Swayne et al. 2009; Bialecka-Fornal et al. 2016), and their excitation and emission wavelengths are summarized in Table 1. For example, the red–orange emitting DsRed (Olenych et al. 2007; Chapuis et al. 2019), and their derivatives like DsRed2, DsRed-Express, DsRed-Express2, mRFP1, and mCherry (Shaner et al. 2004; Olenych et al. 2007; Strack et al. 2008) are popular reporters used extensively in both yeasts and filamentous fungi (Table 1). Although the application of DsRed in various fungi is widespread and successful, there were difficulties with the use of this fluorescent tagging protein including faint emission, incomplete maturation and formation of large protein aggregates inside living cells due to the obligate tetrameric structure of DsRed (Chapuis et al. 2019). This technical challenge stimulated the development of improved and monomeric variants of DsRed (Table 1; https://www.fpbase.org/protein/dsred/; Chapuis et al. 2019). It is noteworthy that protein engineering of slowly maturing, tetrameric DsRed has provided yeast experts with valuable tools to meet the challenges coming from the red fluorescent protein labelling of rapidly growing *S. cerevisiae* cultures (Gavin et al. 2002; Janke et al. 2004). Codon optimization and additional mutations are also frequently carried out to enhance the performance of various fluorescent proteins in yeasts (Van Genechten at el. 2021). For example, the yeast-enhanced mRFP (yEmRFP) is a convenient red emitting marker for genetic studies in *S. cerevisiae* (Keppler-Ross et al. 2008; Misumi et al. 2019), *C. albicans* (Keppler-Ross et al. 2008) and *Kluyveromyces marxianus* (Suzuki et al. 2015). A yEmCherry labelling system is also available for subcellular localization studies in the emerging pathogen *Candida auris* (Defosse et al. 2018). In in vivo assays, yEmRFP was expressed in *C. albicans*, which strain was used in murine macrophage up-take assays (Keppler-Ross et al. 2010). GFP and *C. albicans* codon-adapted RFP (dTOM2) were employed to label *C. albicans* cells in mouse gut colonization assays, which made precise quantification of fungal populations possible via both standard in vitro cultures and flow cytometry (Prieto et al. 2014). Although the phototoxic characteristics of engineered fluorescent proteins were only rarely considered and quantified until recently (Strack et al. 2008), this aspect is gaining ground in fluorescent protein design (Strack et al. 2008, 2009; Lam et al. 2012; Vegh et al. 2014; Bajar et al. 2016; Zhang and Ai 2020; Wu et al. 2021).

**Table 1:** Red, far-red, and near-infrared fluorescent reporter proteins available in mycology (https://www.fpbase.org/)

Protein	Organism	Oligomerization	Excitation *λ*	Emission *λ*
			(nm)	(nm)
DsRed	*Discosoma sp.*	4	558	583
DsRed.T3	*Discosoma sp.*	4	560	587
DsRed.T4	*Discosoma* sp.	4	555	586
DsRed Express	*Discosoma* sp.	4	554	586
DsRed Express2	*Discosoma* sp.	?	554	591
DsRed Max	*Discosoma* sp.	4	561	594
DsRed2	*Discosoma* sp.	4	561	587
tdTomato	*Discosoma* sp.	2	554	581
mRFP1 (mRFP)	*Discosoma* sp.	1	584	607
mCherry	*Discosoma* sp.	1	587	610
mRaspberry	*Discosoma* sp.	1	598	625
mPlum	*Discosoma* sp.	1	590	649
E2-Crimson	*Discosoma* sp.	4	611	646
TagRFP	*Entacmaea quadricolor*	2	555	584
TagRFP-T	*Entacmaea quadricolor*	1	555	584
mRuby2	*Entacmaea quadricolor*	1	559	600
mRuby3	*Entacmaea quadricolor*	1	558	592
Katushka	*Entacmaea quadricolor*	2	588	635
mKate	*Entacmaea quadricolor*	1	588	635
mKate2	*Entacmaea quadricolor*	1	588	633
HcRed	*Heteractis crispa*	4	592	645
iRFP682	*Rhodopseudomonas palustris*	2	663	682
iRFP713 (iRFP)	*Rhodopseudomonas palustris*	2	690	713

Multicolor fluorescent imaging systems including red fluorescent proteins are available for a number of yeasts. For example, mCherry was included in a 3-colour (mTFP1/mCitrine/mCherry) live-cell imaging tool box in budding yeast (Higuchi-Sanabria et al. 2016), and other 3-colour and 4-colour imaging systems with fluorescent proteins also emitting light in the red (TagRFP-T or mRuby2) spectra are available now for *S. cerevisiae* and the fission yeast *Schizosaccharomyces pombe* as well (Bialecka-Fornal et al. 2016). Importantly, TagRFP-T and mRuby2 outperformed mCherry as a red fluorescent protein label in baker’s yeast after assessing and comparing brightness, photostability, and perturbation of tagged proteins (Lee et al. 2013). Red and orange fluorescent proteins (the DsRed derivatives mCherry, mOrange2, and tdTomato) together with the *Entacmaea quadricolor* fluorescent protein monomers TagRFP-T and mKate were systematically tested to tag microtubules in the fission yeast *S. pombe* (Snaith et al. 2010) (Table 1).

A 3-colour imaging toolbox (incorporating mCherry) available for *C. albicans* could also be employed to construct fluorescent fusion proteins in *Candida parapsilosis* (Gonia et al. 2016) and, furthermore, a 3-colour toolbox including mCherry has been developed for *C. glabrata* (Yáñez-Carrillo et al. 2015) and other *Candida* spp. (Gonia et al. 2017). In a novel approach, the Clox marker recycling system was adapted to pFA backbone vectors in *C. albicans*, and the new toolkit contains several fluorescent reporter proteins including mCherry (Dueñas-Santero et al. 2019). A 4-colour fluorescent imaging toolbox incorporating cyan, green, yellow, and red (mCherry) tagging proteins was also developed for the emerging human pathogen yeast *Candida guilliermondii* (Courdavault et al. 2011). In the human pathogenic encapsulated basidiomycetous yeast *Cryptococcus neoformas*, a number of fluorescent reporter proteins are applicable including the red mRuby3 in addition to DsRed and mCherry (Spencer et al. 2020), and mCherry was also incorporated and expressed in the “Safe Heaven 2” gene-free intergenic site in the *C. neoformans* genome (Upadhya et al. 2017).

Considering heterologous protein expression in the methylotrophic yeast *Pichia pastoris*, mCherry and mTFP (a cyan fluorescent protein; https://www.fpbase.org/protein/mtfp/) labelled K28 *S. cerevisiae* killer toxin variants were expressed with good yields and were used in yeast cell binding studies (Giesselmann et al. 2017). The high efficiency of an episomal expression plasmid pPICZαBHF was demonstrated by expression of RFP in *P. pastoris* (Chen et al. 2017), and the efficiency of a hybrid protein secretion signal consisting of the *S. cerevisiae* Ost1 signal sequence and the α-factor pro-region was tested in *P. pastoris* expressing the red fluorescent protein E2-Crimson (Barrero et al. 2018). The CL7-tagged fluorescent proteins sfGFP (superfolder GFP) and mCherry were also used to demonstrate the efficiency of a novel CL7/Im7 ultra-high-affinity-based surface display system in *P. pastoris* (Li et al. 2019a).

Red fluorescent protein reporters are used frequently in industrially and biomedically important filamentous fungi as well. For example, DsRed was expressed in the mesophilic, cellulolytic enzyme producer *Trichoderma reesei* under the control of the promoter of the major cellular gene *cbh1*, and the germinated spores were screened for cellulase hyperproducers by fluorescence-assisted cell sorting (Gao et al. 2018). Engineering of the *T. reesei cbh1* promoter significantly increased DsRed protein expression (Sun et al. 2020), and controlled expression of codon-optimised monomeric superfolder GFP and mCherry from a “soft-landing” site juxtaposed to the *sdi1* succinate dehydrogenase locus may be a helpful tool in studying the secretory pathway in this important cellulose producer fungi (Kilaru et al. 2020). *T. reesei* strains expressing DsRed-tagged endoglucanase, cellobiohydrolase, and β-glucosidase played a valuable role in mapping the localization and dynamics of cellulose production in this industrially important fungus (Li et al. 2019b), and to test a versatile *T. reesei* heterologous protein expression system, RFP and the *Humicola insolens egv3* alkaline endoglucanase reporter genes were expressed (Zhang et al. 2018). Conidiospores of the opportunistic human pathogenic fungus *A. fumigatus* were DsRed-labelled and employed in an immunosuppressed mouse model to test the clearance of this pathogen by ultrashort cationic lipopeptides (Vallon-Eberhard et al. 2008). Furthermore, dual labelling of *A. fumigatus* conidia with DsRed (expressed constitutively only in live conidia) and Alexa Fluor 633 dye (it stains both live and dead cells) allowed the researchers to study the interactions between human primary immune cells and *A. fumigatus* in vitro (Brunel et al. 2017). RFP-expressing *A. fumigatus* was constructed and used to gain a deeper insight in the pathogenesis of *A. fumigatus* keratitis (Leal Jr. et al. 2010).

Considering plant pathogenic fungi, signal intensity and bleaching were compared for the red fluorescent proteins mRFP, TagRFP, mCherry, and tdTomato in *Zymoseptoria tritici* (a wheat pathogen) both *in* and *ex planta* (Schuster et al. 2015). While mCherry gave the highest intensity signal, it bleached fast and, hence, mRFP was recommended for long-term observation experiments (Schuster et al. 2015). Red fluorescent protein reporters (DsRed, DsRed2, DsRed-Express, mRFP1, tdTomato, and mCherry) have also been used in a number of studies to visualize infecting hyphae of plant pathogenic fungi penetrating various plant tissues. Recent publications in this field include *Penicillium digitatum* (on citrus fruit, DsRed; Vu et al. 2018), *Penicillium rubens* (on tomato roots, DsRed; Vallarino et al. 2018), *Verticillium dahlia* (on tobacco seedlings, mCherry; Su et al. 2018), and *Epichloȅ* strains (on ryegrass seedlings, DsRed; Hettiarachchige et al. 2019). Furthermore, DsRed-labelled *Verticillium longisporum* was used to test the hypothesis that disease transmission may occur by seeds from European winter oilseed rape production (Zheng et al. 2019), and the role of arthropod vectors in the propagation of grapevine trunk disease pathogenic fungi was demonstrated by the application of DsRed-Express tagged *Phaeomoniella chlamydospora* (Moyo et al. 2014). GFP and DsRed expressing endophytes like *Diaporthe schini* seem to be especially useful when these strains are tracked in their natural environments (Felber et al. 2019). It is important to note that interference with autofluorescence of chlorophylls and other cell constituents may mask GFP fluorescence in above-ground photosynthesizing plant tissues (Zhou et al. 2005; Berg and Beachy 2008). Meanwhile, the interference between chlorophylls and red fluorescent proteins can be minimalized by suitable filter sets (Jach et al. 2001; Okwuonu et al. 2015) or can be resolved by spectral unmixing techniques (Berg and Beachy 2008).

### Far-red and near-infrared fluorescent protein probes

To decrease phototoxicity further and facilitate deep-tissue analyses, a new set of far-red emitting fluorescent proteins has been developed with red-shifted excitation wavelengths (Table 1), and transmission imaging performed at wavelengths in the near-infrared spectrum is gaining ground in Mycology.

The fluorescent protein mPlum with far-red emission was evolved in a mammalian B cell-line using somatic hypermutation (Wang and Tsien 2006) producing a red fluorescent protein variant mRFP1.2 (Table 1), which can be incorporated into 3-colour and 4-colour imaging systems recommended for use in budding yeast (Bialecka-Fornal et al. 2016). Whole-body imaging in small laboratory animals required the development of further fluorescent proteins emitting in the far-red spectrum with high brightness and photostability like Katushka, mKate, and mKate2 (Table 1) (Shcherbo et al. 2009). After thorough optimization of illumination and other experimental conditions, Katushka, mRaspberry, and mCherry proved to be highly suitable for deep-tissue molecular imaging applications (Deliolanis et al. 2008). When applied together with a lipophilic fluorescent dye, Katushka was successfully used to track multiple bacterial strains in the intestine of mouse both temporally and spatially in 3D by fluorescence molecular tomography (Peñate-Medina et al. 2019).

In response to increasing demands for even further red-shifted probes, a novel phytochrome-based near-infrared fluorescent protein, iRFP (iRFP713; Table 1), characterized with higher effective brightness, intracellular stability, and photostability than earlier phytochrome-derived fluorescent probes was produced for in vivo imaging (Filonov et al. 2011). Later, together with other fluorescent proteins (yemCFP, sfGFP, mCitrine, mCherry), the tandem dimer iRFP (tdiRFP) was incorporated in a set of budding yeast single integration vectors replacing the entire deficient auxotrophy marker locus and used as gene tagging plasmids, ideally for tagging abundant proteins (Wosika et al. 2016).

The near-infrared fluorescent protein iRFP713 (Table 1) was expressed in lactobacilli and *E. coli* that were visualized in the intestine of living mice using epifluorescence imaging or fluorescence tomography. Another fluorescent protein iRFP682 (Table 1) was also expressed in *Lactobacillus plantarum*, enabling the concomitant detection of two bacterial populations in living mice (Berlec et al. 2015).

The far-red, near-infrared, phytochrome-based fluorescent proteins like phycobiliproteins of cyanobacteria are becoming widely used even in mammalian cells, where biliverdin is typically present and accessible for these proteins (Chernov et al. 2017; Ding et al. 2017, 2018; Hou et al. 2019a, b; Li et al. 2019c). The application of these fluorescent proteins, however, is limited to chromophore producing fungi, e.g. in genetically engineered *S. cerevisiae* (Hochrein et al. 2017).

It is important to note that fungi can differentiate between less and more red shifted (e.g. *λ* ≅ 700 nm and *λ* ≅ 760 nm) illumination with marked alterations in conidiogenesis, germination of conidia, and even in illumination-responsive changes in global gene expression patterns (Yu et al. 2021). In order to facilitate the shift towards the near-infrared spectrum in microscopic techniques used in Mycology, the scope of on-going and future fungal physiological, developmental, and omics-based studies should be expanded to cover the near-infrared spectrum of light as well. Although our knowledge on sensing infrared light by fungi is rather scarce, the involvement of FphA phytochrome in temperature sensing in *A. nidulans* (Yu et al. 2019) warns us that infrared light might not be neutral for fungi either.

## Live-cell imaging, time-lapse, high-resolution, and special microscopies

Red fluorescent proteins are also commonly used to visualize cellular movements and positioning of labelled proteins within live fungal cells. Nevertheless, the intrinsic properties of excitation light limit resolution and may cause photobleaching and photoxicity especially during prolonged image acquisition (Escorcia et al. 2019). To balance optimally resolution and photodamage in answering specific research questions, various advanced microscopy approaches and appropriate model systems have been developed. Advantages and disadvantages of currently applied microscopic techniques are summarized in Table 2.

**Table 2:** Comparison of microscopic techniques used in Mycology

Methods	Information obtained	Advantages	Disadvantages			Description of the method			Application of the method in fungi
**Time-resolved microscopy of cellular processes**									
Time-lapse microscopy, classical	Cellular dynamics	Live cell functions can be followed, e.g., cell division, cell motion	Bleaching limits the time frame of analysis, light intensity might affect cell functions			Mubaid and Brown 2017; Escorcia et al. 2019			Li et al. 2011; Escorcia et al. 2019
Time-lapse microscopy, using far-red and near infrared light	Cellular dynamics	Live cell functions can be followed, e.g., cell division, cell motion; no bleaching, deeper penetration	Application of far-red and near-infrared photons decreases optical resolution			Nagy et al 2014; Talas et al. 2019			Nagy et al. 2014; Talas et al. 2019
**Spatially resolved microscopy**									
Confocal laser scanning microscopy (CLSM), classical	Mostly structural information in 3D	Improved resolution, and contrast, 3D stacks can be analyzed	Special microscopes are needed, time consuming, bleaching may occur			Mouriño-Pérez and Roberson 2015; Bayguinov et al. 2018			Mouriño-Pérez and Roberson 2015; Leiter et al. 2016; Szabó et al. 2020; Maione et al. 2022
Spinning disc confocal microscopy (SDCM)	Live cells monitored at subcellular 3D resolution	Fast image formation	Resolution is less than in classical CLSM, bleaching may occur			Montecchi and Schwobb 2018			Montecchi and Schwobb 2018
Total internal reflection microscopy (TIRF)	Structural information and dynamics, including single molecule tracking	Less background fluorescence, improved z resolution	Special optics are needed, only cell surfaces on substrate can be analyzed			Oheim et al. 2019			Kaksonen et al. 2005
Light sheet fluorescence microscopy (LSFM), incl. selective plane illumination microscopy (SPIM)	Time-resolved live-cell imaging	Less background fluorescence and bleaching, fast image formation, whole live organisms like zebrafish can be analyzed	Special instrumentation is needed, positioning of the sample may be tedious			Langowski 2017			Licea-Rodriguez et al. 2019
**Molecular dynamics and interactions**									
Fluorescence recovery after photobleaching (FRAP)	Molecular dynamics in live cells	Relatively simple mathematical background, semiquantitative analysis possible, immobile molecules are also assessed	Special microscopes (minimally a CLSM) needed	Pearson et al. 2006					Pearson et al. 2006
Fluorescence correlation spectroscopy (FCS)	Molecular dynamics in live cells	Level of aggregation, binding to less mobile components, and photophysical processes can be analyzed, absolute concentration and diffusion constant determined in single measurement	Appropriate algorithms and models should be applied, concentration of the probes cannot be too high, only mobile molecules are decetected and analyzed	Vámosi et al. 2019					Slaughter et al. 2007; Pack et al. 2014
Fluorescence cross-correlation microscopy (FCCS)	Molecular dynamics and associations monitored in live cells	As in FCS Stable molecular associations can be revealed	As in FCS. Optically aligned dual excitation and good spectral separation of emission is needed	Vámosi et al. 2019					Slaughter et al. 2007; Pack et al. 2014
Förster resonance energy transfer (FRET)	Molecular interactions	Regular fluorescence microscopes can be used	Special probe pairs are needed, appropriate algorithms should be applied, stability of association not assessed	Vereb et al. 2004; Roszik et al. 2008					Slaughter et al. 2007; Skruzny et al. 2019
Raster image correlation spectroscopy (RICS)	Molecular diffusion and aggregation	Sensitive analysis of various levels of molecular clustering	Appropriate algorithms and models should be applied	Moreno and Aldea 2019﻿					Whiteside et al. 2012; Moreno and Aldea 2019; van’t Padje et al. 2021
**Super-resolution microscopy (SRM)**					Tosheva et al. 2020; Schermelleh et al. 2019			Dodgson et al. 2015; Wollman and Leake 2015	
Photoactivated localisation microscopy (PALM)	Superfine structural information	Very high optical resolution	Special fluorescence probes are needed in low concentration, time consuming, special software is needed, no dynamic information		Betzig et al. 2006; Henriques et al. 2011			Gao et al. 2021b	
Stochastic optical reconstruction microscopy (STORM)	Superfine structural information	Very high optical resolution can be achieved, 3D version available	Photo-switchable probes are needed, time consuming, special software is needed, no dynamic information		Rust et al. 2006; Henriques et al. 2011			Lin et al. 2016	
Structured illumination microscopy (SIM)	Superfine structural information	Very high optical resolution can be achieved, less emitted light is discarded, shorter image acquisition time, time-lapse imaging possible	Special optical illumination system and special software needed		Gustafsson 2000; Komis et al. 2014			Dodgson et al. 2015; Götz et al. 2020; Chen et al. 2021	
Stimulated emission depletion (STED) microscopy	Superfine structural information	Very high resolution can be achieved in 3D	Expensive, high power lasers and special fluorescence probes are needed, slow data collection, no dynamic information		Hell and Wichmann 1994			Dodgson et al. 2015; Götz et al. 2020; Chen et al. 2021	
Expansion microscopy (ExM)	Superfine structural information	Regular fluoresceence microscopy can be used, high resolution (4–10 times) can be achieved, can be combined with any SRM	Cell walls should be digested, distortion should be avoided using tetrahedron like monomers and appropriate control experiments, no dynamic information		Chen et al. 2015; Truckenbrodt et al. 2018; Gao et al. 2021a;			Götz et al. 2020; Chen et al. 2021; Korovesi et al. 2022	
**Multiphoton microscopy**	Structural and dynamic information	Deep penetration, good resolution, no bleaching, less fluorescence background	Expensive lasers and optics are needed		Chapuis et al. 2019			Bago et al. 1999; Lee et al. 2018; Câmara et al. 2019	

### Time-resolved microscopy of cellular processes

Phototoxicity can be countered by not exposing cells to short wavelengths for too long or too frequently in time-lapse microscopy. In fission yeast, the proper selection of illumination conditions, strains, and fluorescence markers made possible the examination of nuclear dynamics during mitotic cycles and meiosis (Escorcia et al. 2019). In this study, chromosome dynamics (Hht1-mRFP or Hht1-GFP) and segregation (Sad1-DsRed), cytoskeleton dynamics (Atb2-mRFP), transcriptional activation (Tos4-GFP), and cohesion stability in meiosis (Rec8-GFP) could all be monitored using the reporter proteins indicated in parentheses (Escorcia et al. 2019). Chromosome dynamics was also tracked in live *S. cerevisiae* cells using fluorescent proteins. For example, arrays of tet and lac operators were inserted into each repeat of the two rDNA homologs in budding yeast and the operators were visualized and tracked by time-lapse 3D fluorescence microscopy via the expression of tet-repressor-fused GFP and lac-repressor-fused RFP (Li et al. 2011).

The Rosella biosensor system based on the fast-maturing pH-stable DsRed.T3 (Table 1) fused with the green ratiometric protein pHluorin (https://www.fpbase.org/protein/phluorin-ratiometric/) was constructed to study the vacuolar turnover of cytosol and organelles and successfully employed to visualize nitrogen-starvation induced mitophagy in *S. cerevisiae* (Mijaljica et al. 2011)*.*

Caspofungin exposure triggered rapid phosphatidylinositol 4,5-bisphosphate [PI(4,5)P2]-septin and protein kinase C-Mkc1 response in *C. albicans*. The PI(4,5)P2-septin responses were recorded in live-cell microscopy by expressing a GFP-pleckstrin homology domain, which binds PI(4,5)P2, and the RFP-septin fusion proteins in both wild-type and *mkc1* mutant cells (Badrane et al. 2016).

GFP and mCherry labelling were also employed to study spatiotemporal protein–protein interactions in single yeast cells after fusing a-mating type and α-mating type *S. cerevisiae* cells using two-channel fluorescence time-lapse microscopy with the SPLIFF method. In this approach, one protein of interest is attached to a linear mCherry-C_ub_ (the C-terminal half of ubiquitin)-GFP fusion in one mating type and the other protein is fused to the N-terminal half of ubiquitin (N_ub_) in the other mating type. After mating, if the two proteins of interest interact, the two halves of ubiquitin complement each other, which causes, the GFP to be cleaved and degraded. As a result, the originally balanced red-to-green fluorescence ratio will shift towards the red marking in space and time where the two proteins have interacted (Dünkler et al. 2015).

Tandem fluorescent protein timers are constructed via the combination of faster maturing enhanced superfolder GFP and slower-maturing mCherry. Such constructs are applicable to study protein turnover and trafficking e.g. in *S. cerevisiae* cells but a special care should be taken of the proteasomal degradation of these dimers (Khmelinskii et al. 2016). A dynamic protein synthesis translocation reporter (dPSTR) was designed to avoid problems arising from the slow maturation of fluorescent proteins. dPSTR consists of two transcriptional units the first of which constitutively expresses a fluorescent protein freely diffusing between the cytosol and the nuclei. The second unit incorporates two nuclear localization signals (NLSs) driven by the promoter under investigation. Both these units also contain a synthetic bZip domain. The activated promoter driving the synthesis of the NLS-bZIP fusion protein instantaneously disrupts the equilibrium of the already matured FP-bZIP fusion protein, causing the increase of its presence in the nucleus, proportionally to the activity of the promoter in each individual cell (Aymoz et al. 2016).

To decrease the general phototoxicity of visible light in time-lapse microscopy, diodes emitting light in the infrared spectrum (LED, *λ* = 940 nm) were introduced by Nagy et al. (2014) to monitor and quantify the adherence time, hyphal outgrowth time, and hyphal growth rate of *C. albicans* cultured in RPMI plus 10% foetal bovine serum in a CO_2_ incubator. The system was highly suitable for quantifying the effects of tyrosol and farnesol treatments as well as *Cappz1* and *hgc1* mutations on cell motility, morphological transition, and hyphal growth rate (Nagy et al. 2014). Later the same group used similar experimental arrangements to monitor the effects of the antimycotics amphotericin B and voriconazole on the adherence and germination of *A. fumigatus* conidia and the growth dynamics (elongation and branching) of *A. fumigatus* hyphae (Talas et al. 2019).

### Spatially resolved microscopy

Long-term and frequent illumination of fluorescent proteins may cause radiation-dependent DNA damage. Responses to such DNA damage were monitored expressing Rad52-GFP (Rad52 is a DNA double strand break repair protein) and mCherry-alpha-tubulin in *S. cerevisiae* and the persistence of Rad52-GFP fluorescence clearly depended on the 488 nm excitation light dose applying spinning disk confocal microscopy (SDCM). Interestingly, the topoisomerase 1 inhibitor camptothecin resulted in extended cell cycle and persistent Rad52-GFP fluorescence, and a *mec1Δ sml1 S. cerevisiae* strain defective in DNA damage-elicited cell-cycle arrest showed persistent Rad52-GFP fluorescence in the presence of camptothecin and underwent cell death a few cycles later (Montecchi and Schwob 2018).

In SDCM, rather than using a single pinhole as in classical confocal laser scanning microscopy (CLSM), hundreds of pinholes arranged in spirals are applied on an opaque disk, which rotates at high speeds. This arrangement enables fast image acquisition and reduces the excitation energy, which mitigates phototoxicity on the observed species as well as reduces the rate of photobleaching of the applied fluorophores. This renders SDCM a system of choice for microscopic observation of living cells or organisms. Although the resolution is not as good as in CLSM because a bit less out-of-focus light is removed in the SDCM system than in CLSM, the increase in acquisition speed presents an advantage worth this compromise (Bayguinov et al 2018).

Total internal reflection fluorescence (TIRF, Oheim et al. 2019) microscopy provides a mechanism that can limit the region where the fluorophores are excited (and therefore detected) to a thin section of the sample, thereby eliminating unwanted background fluorescence, which would emanate from other planes when using conventional illumination. This increases the signal-to-noise ratio, and improves z-resolution. The principle behind TIRF microscopy is the exploitation of the critical angle at which light beams impinging on the surface of an optically less dense medium (e.g. entering from the coverslip into the cell) cannot penetrate into this medium. Beyond this angle, light beams are reflected from the border of the two substances; however, an evanescent electromagnetic wave penetrates into the less dense medium, with an intensity that decreases exponentially. This fast decay allows the excitation of only those fluorophores that are within a few hundred nanometers to the surface. Thus, TIRF microscopy is most often utilized to monitor events at the plasma membrane such as endocytic processes. A good example for this is the study of Kaksonen et al. (2005) where GFP-labelled clathrin and mRFP-tagged actin marker protein Abp1p were expressed and visualized in vivo in *S. cerevisiae*.

More recently, multicolour light-sheet fluorescence microscopy (LSFM) systems were developed using static illumination light-sheets and decoupling the excitation and detection optical paths. Here, the planar illumination causes less photodamage and allows faster wide-field imaging with high-efficiency cameras while minimizing out-of-focus fluorescence. One version of LSFM, selective plane illumination microscopy (SPIM, Langowski 2017) was applied to detect GFP-tagged microtubules and RFP-labelled nuclei simultaneously in growing *N. crassa* hyphae. Time-lapse images were merged to show the dynamics of these structures in the apices of living hyphae. The system was suitable for the rapid and simultaneous acquisition of multifluorescence images, which were useful in three-dimensional imaging of hyphal structures (Licea-Rodriguez et al. 2019).

### Molecular dynamics and interactions

Time-resolved fluorescence recovery after photobleaching (FRAP) combined with model convolution microscopy was employed to measure nanometer scale gradients in spindle microtubule dynamics in budding yeast monitoring the fluorescence of GFP fused to Tub1 (tubulin marker); meanwhile, Spc29-RFP was used to label spindle pole bodies (Pearson et al. 2006). F-actin dynamics was first studied in *N. crassa* by visualizing actin using the Lifeact system incorporating a GFP- or TagRFP-labelled 17 amino acid peptide of *S. cerevisiae* Abp140p actin-binding protein (Berepiki et al. 2010). Time-lapse imaging and the expression of GFP- and mCherry (optionally tdTomato)-labelled formins (For3, Cdc12), formin fusions (Tea1-For3, Tea1-Cdc12), and myosin were employed to check the functional behaviour of actin filaments (dynamics, actin-binding protein, and myosin motor activities) modulated by formins in fission yeast (Johnson et al. 2014).

Fluorescence correlation spectroscopy setups like one-colour fluorescence auto-correlation (FCS, Vámosi et al. 2019) and dual-colour cross-correlation (FCCS) microscopies based on GFP- and mCherry-labelled proteasome proteins played a predominant role in shedding light on the spatio-temporal dynamics and cytoplasmic assembly of the 26S proteasome in live budding yeast (Pack et al. 2014). FCS is based on monitoring fluctuation in fluorescence intensity detected from a tiny volume determined by the confocal illumination. The obtained fluorescence auto-correlation function allows the measurement of the concentration of fluorescent molecules and the mobility parameter of these molecules reflecting their molecular size. Dual-colour FCCS provides a cross-correlation function and two auto-correlation functions allowing the measurement of direct interaction, and stable co-mobility of two spectrally distinct (e.g. green and red) fluorescent molecules transiting through the observation volume. Using this approach, the authors revealed that 26S proteasome completes its assembly process in the cytoplasm and translocates into the nucleus through the nuclear pore complex as a holoenzyme.

GFP and mCherry fluorescent proteins were successfully employed to set up live-cell FCS and FCCS, complemented with FRET (Förster (fluorescence) resonance energy transfer, Vereb et al 2004) protocols to map dynamic protein interactions between elements of the Fus3 mitogen-activated protein kinase signalling pathway in *S. cerevisiae* (Slaughter et al. 2007). The authors detected interaction between Ste5 and Fus3 by FCCS and they wanted to confirm this interaction by FRET measurement. Significant FRET efficiency was detected between the donor (GFP) and acceptor (mStrawberry) labelled proteins using the acceptor photobleaching version of FRET methods (Roszik et al 2008). In this method, the increase in donor fluorescence intensity after acceptor photobleaching was utilized to calculate FRET efficiency, which proved that the Ste5 and Fus3 proteins are within 10 nm in the shmoo tip in the yeast cell.

In a more recent study, transient interactions between Ydj1-GFP and Ssa1-mCherry fluorescent protein–labelled chaperones were investigated in live budding yeast cells using raster image correlation spectroscopy (RICS) (Moreno and Aldea 2019). RICS is a spatial variant of fluctuation microscopies in which the sample is repeatedly raster scanned using appropriately fine-tuned scan and pixel times that enable the revelation of intensity changes in pixels occurring on the same timescale. From the data, both the diffusion coefficient and concentration of the mobile species can be extracted.

### Super-resolution microscopy (SRM)

An imaging tool based on super-resolution localization microscopy (SRM, Tosheva et al. 2020) with millisecond time resolution, convolution analysis, and automated image segmentation was developed to determine GFP-tagged and mCherry-tagged proteins in single functional *S. cerevisiae* cells (Wollman and Leake 2015). Super-resolution microscopy has many modalities. Of these, stochastic optical reconstruction microscopy (STORM) and photoactivated localisation microscopy (PALM) exploit the possibility of exciting and observing the emission from only a few spatially distant molecules and fitting the point-spread-function (PSF) on the cumulated, spatially spread emitted photons. In another variant, structured illumination microscopy (SIM), the cumulative information from diverse illumination patterns is analyzed to obtain resolution beyond the Abbe limit. Finally, stimulated emission depletion (STED) microscopy reduces the observation volume by using beam shaping optics to project strong laser light in the spectral domain of the expected emission to only the outer regions of the excited confocal volume to cause immediate relaxation in those areas, thereafter detecting the spontaneous emission from the fluorophores remaining excited in the central, considerably smaller region.

Expansion microscopy (ExM, Chen et al. 2015) performed on *U. maydis* sporidia expressing fluorescent fungal rhodopsins or on hyphae of *F. oxysporum* expressing histone H1-mCherry and Lifeact-sGFP and *A. fumigatus* expressing mRFP targeted to mitochondria resulted in about 30 nm spatial resolution of details of subcellular structures (Götz et al. 2020). ExM is a cheap alternative providing quasy-super-resolution images but using a conventional fluorescence microscope. In ExM, cells are fixed and immunostained before amino groups are modified by glutaraldehyde and then the sample is soaked in a monomer solution, which is consecutively polymerized (gelated). Then, the gel is isotropically expanded in water to uniformly extend the distances four to ten times between fluorophores. The sample is analyzed by fluorescence confocal microscopy at its regularly provided resolution; however, the preceding isotropic physical expansion of the specimen still allows bypassing the diffraction limit. Distortion and differences in the specific expansion factors of different organelles could be a problem; therefore, control experiments of the same structure before and after the expansion are required. In addition, the application of modern tetrahedron like monomers makes the expansion highly isotropic (Gao et al. 2021a). This method is difficult to apply to fungi, mainly due to their complex cell wall structure. Götz et al. (2020) successfully modified the method to be applicable to fungal cells using treatment with cell wall degrading enzymes before the fixation step. The authors have demonstrated that ExM with this modification is well suited for studying fungi at sub-diffraction resolution using conventional and confocal laser scanning fluorescence microscopes.

### Multiphoton microscopy for greater penetration depth

Furthermore, two-photon microscopy techniques are becoming popular tools for visualizing subcellular events in fungi and also hyphal structures and their complex interactions with other cells.

Multiphoton microscopy can take advantage of the excellent penetration of red and infrared light in deep tissue (up to 1 mm) complemented with the good visibility and brightness of conventional fluorophores emitting in the visible range (Chapuis et al. 2019). Moreover, since the excitation of the convential fluorophores happens only in a very thin layer of the sample, where the flux of the photons is high enough for simultaneous absorption of two photons, the bleaching and phototoxic effects are negligible for the whole sample. Two-photon microscopy techniques were successfully used to track the localization of nuclei in hyphae of arbuscular mycorrhizal fungi (Bago et al. 1999), to monitor the hyphal distribution of GFP-tagged reporter proteins, and to visualize the orientation of hyphae in a calcofluor-stained catheter model of *C. albicans* biofilm (Zhao et al. 2006). Similar microscopic methods were also employed to study (i) the spatial positioning and elongation of *S. pombe* mitotic spindle (Tolić-Nørrelykke et al. 2004), (ii) the in vivo ribosomal stalk heterogeneity of *S. cerevisiae* (García-Marcos et al. 2008), (iii) the architecture of developing multicellular budding yeast colony (Váchová et al. 2009), and (iv) the progression of allyl alcohol, diallyl disulphide, and garlic (*Allium sativum*) extract induced oxidative stress in *C. albicans* (Lemar et al. 2005, 2007). Cells of the non-*Saccharomyces* yeast species *Torulaspora delbrueckii*, *Metschnikowia pulcherrima*, and *Lachancea thermotolerans* tolerated better dehydration stress when they were enriched in glutathione or trehalose as analyzed by flow cytometry and two-photon laser scanning microscopy (Câmara et al. 2019).

Two-photon microscopy can also help mycologists to reveal the subcellular localization of melanin, NAD(P)H, and protein-bound NAD(P)H in spores (*Aspergillus ochraceus*; Herbrich et al. 2012), and to estimate the metabolic state of *Agaricus bisporus* (Knaus et al. 2013). The uptake of phenanthrene by the white-rot fungus *Phanerochaete chrysosporium* was also followed by this technique (Gu et al. 2016).

Laser power-dependence of photo-oxidation was studied in *S. cerevisiae* by two-photon microscopy (*λ* = 830 nm) as well (Grangeteau et al. 2018). Fungal destruction (plasma membrane permeabilization, cell necrosis) was initiated easily using high-density light flux, which may facilitate the development of future photo-oxidation-based antifungal therapies (Grangeteau et al. 2018).

3D imaging of temporal and spatial development of haustoria on host plants can also be studied by two-photon microscopy (e.g. during the infection of wheat with the rust fungus *Puccinia striiformis*; Sørensen et al. 2012). Moreover, the interaction of the antimicrobial hairpin-like protein EcAMP1 with *Fusarium solani* conidia was also studied by two-photon microscopy (Vasilchenko et al. 2016).

Fine cellular details of animal-fungal pathogen interactions can also be visualized and studied by two-photon microscopy on thick viable lung slices (Hasenberg et al. 2011) or in intact lungs (Bruns et al. 2010) of mice infected by *A. fumigatus.* Using two-photon second harmonic generation microscopy, Lee et al. (2018) were able to detect *C. albicans* and *A. fumigatus* cells in infected corneas in a fungal keratitis rabbit model ex vivo.

## Conclusions, future tendencies

The last decades have seen a vast expansion of molecular biology toolsets applying various fluorescent proteins for observing cells at all levels—starting from single molecule observation and manipulation, through observing molecular interactions in situ, to showing morphological and functional changes at the microscopic level in vitro and in vivo. At the same time, microscopy itself has undergone a spectacular development, bypassing the diffraction limit in several different and complementary ways, and bringing high-resolution and/or highly specialized imaging modalities to everyday use. Mycologists have been at the forefront of many of these developments, and have ingeniously adapted others to the fungi of their interest. However, it has also become clear that fungi have photosensors and react to various visible wavelengths of illumination, thereby altering multiple aspects of their behaviour as an unwanted side effect of microscopic observation. Consequently, new ways of microscopic imaging are necessary to avoid illumination-derived artefacts. One obvious route is to shift wavelengths away from those most efficiently detected by fungi. Conveniently, based on the success of red fluorescent proteins, the novel far-red emitting variants are now gaining space, and some (very) near infrared species are also being considered. In addition, for long period transmitted light microscopy, NIR has appeared on the palette, allowing further reduction in the unwanted effects of illumination. Detection of longer wavelengths, however, has its drawbacks, both in terms of sensor quantum efficiency and optical resolution. One-way around is to use two-photon excitation, which eliminates the visible spectrum from the illumination, which represents the greatest portion of exposure, yet allows high-resolution detection of the emitted visible photons. Given the special technical requirements for this approach, other new developments are also needed to achieve a fine balance between reducing phototoxicity and achieving acceptable resolution and signal-to-noise ratio. These goals could be well aided by recent and ongoing efforts in improving resolution as well as optimizing the use of information in special microscopy approaches such as FRAP, FCS, FRET, RICS, and ExM.
